# An analysis of whether sleep disorder will result in postpartum depression

**DOI:** 10.18632/oncotarget.25219

**Published:** 2018-05-18

**Authors:** Tu-Chen Chung, Chi-Hsiang Chung, Hsuan-Ju Peng, Chang-Huei Tsao, Wu-Chien Chien, Huey-Fang Sun

**Affiliations:** ^1^ Graduate Institute of Medical Sciences, National Defense Medical Center, Taipei, Taiwan; ^2^ School of Public Health, National Defense Medical Center, Taipei, Taiwan; ^3^ Department of Nursing, Taoyuan Armed Forces General Hospital, Taoyuan, Taiwan; ^4^ Department of Medical Research, Tri-Service General Hospital and Department of Microbiology and Immunology, National Defense Medical Center, Taipei, Taiwan; ^5^ National Defense Medical Center, Tri-Service General Hospital, Department of Medical Research, Taipei, Taiwan; ^6^ National Defense Medical Center, School of Nursing, Taipei, Taiwan

**Keywords:** sleep disorder, postpartum depression, national health insurance research database (NHIRD)

## Abstract

Postpartum depression has become an important topic of concern in recent years. However, very few studies on the causes of postpartum depression exist, and the effects of prenatal sleep disorders on the development of postpartum depression among pregnant women have not been elucidated. This study aimed to understand the association between prenatal sleep disorders and postpartum depression. The National Health Insurance Research Database of Taiwan (between 2000 and 2010) was used to assess the effects of prenatal sleep disorder on the risk of postpartum depression using Cox regression analyses. Prenatal sleep disorder in pregnant women increased the risk of developing postpartum depression (the risk in the sleep disorder cases was 5.359-fold increased compared with control cases). In addition, regardless of postpartum week (≤ 6 weeks, 6–12 weeks, or > 12weeks), the risk of developing postpartum depression in pregnant women with prenatal sleep disorders were increased, by 5.461-fold (*P* < 0.001), 3.490-fold (*P* = 0.010), and 3.416-fold (*P* = 0.015), respectively, compared with pregnant women without sleep disorders. Pregnant women with prenatal sleep disorders exhibited increased risks of developing postpartum depression. For pregnant women with prenatal sleep disorder, postpartum intervention measures should be provided as early as possible to reduce the risk of developing postpartum depression.

## INTRODUCTION

Depression has displayed an increasing trend in recent years. Pregnancy increases the chance of developing depression, namely, postpartum depression (PPD). From pregnancy and childbirth to postpartum, women encounter not only enormous physiological changes but also great psychological impacts. The prevalence rates of PPD are approximately 13.8%–19.8% in the USA [1]. and approximately 11.6%–25% in other countries [2–4]. Studies in Taiwan have demonstrated that 36.3%–42.6% of pregnant women in the Taiwan area have PPD symptoms [5–7]. Therefore, the chance of developing PPD is high for women in Taiwan, and this problem requires urgent attention. In the serial editions of Diagnostic and Statistical Manual of Mental Disorders (including DSM-III, DSM-III-R, DSM-IV, DSM-IV-TR, and DSM-5) noted that PPD is defined as the onset of mood disorders starting at postpartum 4 weeks. At least 5 symptoms appear every day: depressed mood, obvious lack of interest in usual activities, a decrease or increase in body weight, insomnia or excessive sleep, slow action or increased activities due to uneasiness, fatigue or decreased energy, feelings of worthlessness or guilt, diminished abilities of thinking or concentration, and occurrence of thoughts of suicide. These symptoms persist for more than two weeks [8, 9]. Some scholars consider that in addition to the mental illness diagnosis and the presentation of symptoms described in the statistical manual, PPD should also cover self-consciousness of being incapable of the roles, functions, and responsibilities of motherhood, feeling guilty, using more invasive or negative methods in child-rearing (even having thoughts and behaviors of hurting the child when having problems with one's parenting style after birth), changes in the spousal relationship, and the inability to concentrate on routine matters [10–13]. Therefore, identifying methods to reduce the development of PPD is very important.

Studies on PPD have increased in number in the past twenty to thirty years, with most studies focusing on risk factors. A few studies have noted that poor postpartum sleep quality can easily increase the risk of PPD [14, 15]. Sleep troubles in pregnant women not only affect their ability to fulfill their multiple postpartum roles but also negatively influence the development of the parent-child relationship [16, 17]. Current PPD studies in Taiwan mainly focus on nursing care; most studies have investigated the effects of PPD on the quality of life of women from psychological, family, and social perspectives [18–20]. Although numerous studies on sleep quality in Taiwan have been reported, few studies have investigated sleep disorders (SD) using pregnant women as the study subjects.

Currently, few reports on sleep assessment targeting pregnant women during pregnancy exist, and no systemic studies have assessed the association between SD and PPD. Therefore, we used the National Health Insurance Research Database (NHIRD) of Taiwan to perform a follow-up study on whether prenatal SD can increase the development of PPD.

## RESULTS

Our study presents that there is no Statistical significance in age, number of births, comorbidities and insured premium both in subjects and control groups. (data not shown).

**Figure 1:**
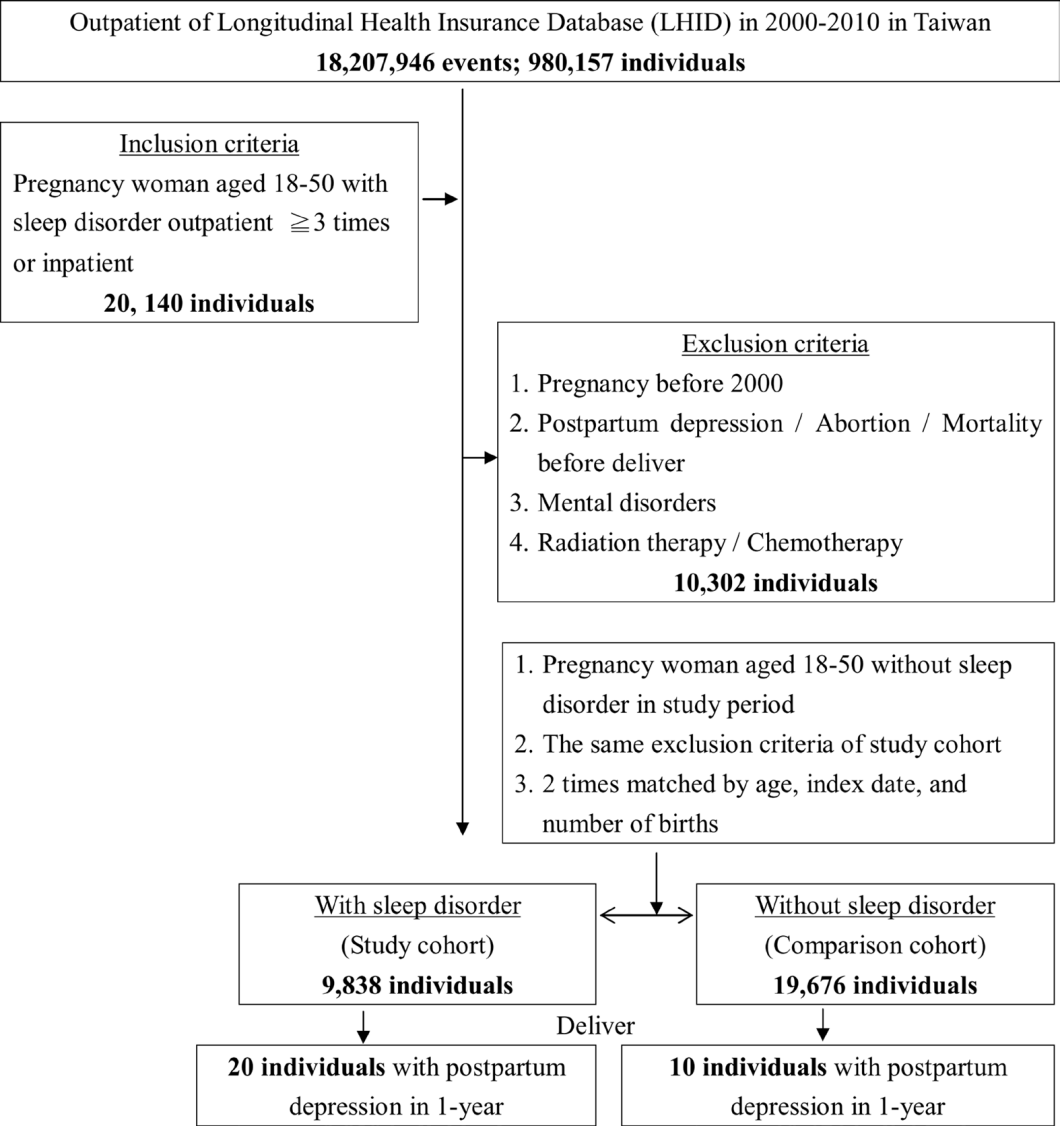
The flowchart of study sample selection from National Health Insurance Research Database in Taiwan

**Figure 2:**
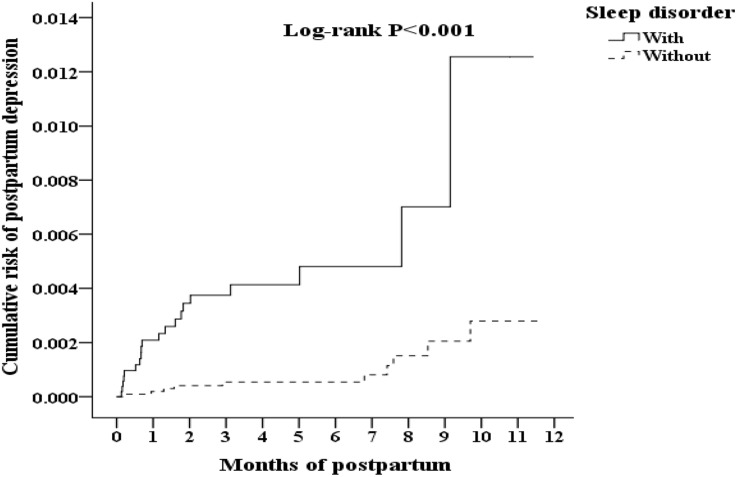
Kaplan–Meier for cumulative risk of postpartum depression among pregnancy woman aged 18–50 after deliver in 1-year tracking stratified by sleep disorder with log-rank test

Figure 1 presents the flowchart for the study sample selection (inclusion and exclusion), follow-up results and the cumulative risks of developing PPD in these two groups (pregnant women with/without SD). Among 1 million individuals, 980,157 had medical records (outpatient, emergency, or inpatient). In total, 20,140 pregnant women with SD were identified. After employing the exclusion conditions (10,302 were excluded), a total of 9,838 pregnant women with SD (19,676 controls were selected using the 1:2 ratio) were included in this study. Of these women, 0.20% developed PPD (20/9,838 people). The percentage of women who developed PPD in the control group (pregnant women without SD) was 0.05% (10/19,676 people). The difference between these two groups reached significance (log-rank *P* < 0.001). Thus, the chance (i.e., risk) of developing PPD in pregnant women with SD was significantly increased compared with pregnant women without SD (Figure 2).

Table 1 presents the distribution of the basic characteristics of the 29,514 cases overall (subject group: 9,838 pregnant women with SD; control group: 19,676 pregnant women without SD) at the follow-up endpoint. The percentage of women who developed PPD in the subject group was significantly increased compared with the control group (0.20% vs. 0.05%; *P* < 0.001).

**Table 1:** Characteristics of study at the endpoint

Sleep disorder	Total		With		Without		*P*
Variables	*n*	%	*n*	%	*n*	%	
**Total**	29,514		9,838	33.33	19,676	66.67	
**Postpartum depression**							< 0.001
Without	29,484	99.90	9,818	99.80	19,666	99.95	
With	30	0.10	20	0.20	10	0.05	
**Age (years)**	35.50 ± 7.32		35.73 ± 7.05		35.25 ± 7.43		0.069
**Age group (years)**							0.167
≦ 20	997	3.38	332	3.37	665	3.38	
21–30	7,309	24.76	2,437	24.77	4,872	24.76	
31–34	6,406	21.70	2,135	21.70	4,271	21.71	
35–37	4,560	15.45	1,520	15.45	3,040	15.45	
38–40	5,373	18.20	1,794	18.24	3,579	18.19	
≧ 41	4,869	16.50	1,620	16.47	3,249	16.51	
**Number of births**	1.82 ± 1.77		1.82 ± 1.80		1.82 ± 1.74		0.548
**Number of births**							0.999
1 (First-time birth)	19,161	64.92	6,387	64.92	12,774	64.92	
≧ 2	10,353	35.08	3,451	35.08	6,902	35.08	
**Hypertension**							< 0.001
Without	28,254	95.73	9,217	93.69	19,037	96.75	
With	1,260	4.27	621	6.31	639	3.25	
**DM**							< 0.001
Without	28,519	96.63	9,380	95.34	19,139	97.27	
With	995	3.37	458	4.66	537	2.73	
**Hyperlipidemia**							< 0.001
Without	29,153	98.78	9,634	97.93	19,519	99.20	
With	361	1.22	204	2.07	157	0.80	
**COPD**							< 0.001
Without	28,944	98.07	9,457	96.13	19,487	99.04	
With	570	1.93	381	3.87	189	0.96	
**CKD**							0.216
Without	29,318	99.34	9,767	99.28	19,551	99.36	
With	196	0.66	71	0.72	125	0.64	
**IHD**							< 0.001
Without	28,928	98.01	9,576	97.34	19,352	98.35	
With	586	1.99	262	2.66	324	1.65	
**CHD**							0.009
Without	29,375	99.53	9,778	99.39	19,597	99.60	
With	139	0.47	60	0.61	79	0.40	
**Stroke**							< 0.001
Without	29,139	98.73	9,633	97.92	19,506	99.14	
With	375	1.27	205	2.08	170	0.86	
**Cancer**							0.999
Without	29,490	99.92	9,830	99.92	19,660	99.92	
With	24	0.08	8	0.08	16	0.08	
**Anxiety**							0.032
Without	28,925	98.00	9,620	97.78	19,305	98.11	
With	589	2.00	218	2.22	371	1.89	
**Depression**							< 0.001
Without	28,952	98.10	9,455	96.11	19,497	99.09	
With	562	1.90	383	3.89	179	0.91	
**Obesity**							< 0.001
Without	29,480	99.88	9,817	99.79	19,663	99.93	
With	34	0.12	21	0.21	13	0.07	
**Season**							< 0.001
Spring (March-May)	6,978	23.64	2,197	22.33	4,781	24.30	
Summer (June-August)	7,580	25.68	2,499	25.40	5,081	25.82	
Autumn (September-November)	8,021	27.18	2,907	29.55	5,114	25.99	
Winter (December-Feburary)	6,935	23.50	2,235	22.72	4,700	23.89	
**Urbanization level**							< 0.001
1 (The highest)	10,267	34.79	3,079	31.30	7,188	36.53	
2	12,327	41.77	3,992	40.58	8,335	42.36	
3	2,671	9.05	982	9.98	1,689	8.58	
4 (The lowest)	4,249	14.40	1,785	18.14	2,464	12.52	
**Insured premium (NT$)**							0.068
< 18,000	22,712	76.88	7,600	77.02	15,112	76.80	
18,000–34,999	6,609	22.37	2,193	22.22	4,416	22.44	
≧ 35,000	223	0.75	75	0.76	148	0.75	

*P*-value (category variable: Chi-square/Fisher exact test; continue variable: *t*-test).

The percentages of the development of comorbidities, including hypertension (6.31% vs. 3.25%; *P* < 0.001), DM (4.66% vs. 2.73%; *P* < 0.001), hyperlipidemia (2.07% vs. 0.80%; *P* < 0.001), COPD (3.87% vs. 0.96%; *P* < 0.001), IHD (2.66% vs. 1.65%; *P* < 0.001), CHD (0.61% vs. 0.40%; *P* = 0.009), stroke (2.08% vs. 0.86%; *P* < 0.001), anxiety (2.22% vs. 1.89%; *P* = 0.032), depression (3.89% vs. 0.91%; *P* < 0.001), and obesity (0.21% vs. 0.07%; *P* < 0.001) in the subject group were significantly increased compared with the control group. The percentage of individuals in the subject group who developed PPD in autumn (29.55% vs. 25.99%) was significantly increased compared with the control group (*P* < 0.001). The percentages of level 3 (9.98% vs. 8.58%) and level 4 (18.14% vs. 12.52%) urbanization of residence in the subject group were increased compared with the control group (*P* < 0.001).

Table 2 lists the univariate and multivariate analysis results on the influencing factors of PPD. After comorbidity, season, urbanization level, and insured premium (income) were controlled, the chance (risk) of developing PPD in pregnant women with SD was 5.359-fold (*P* < 0.001) increased compared with the control group (pregnant women without SD).

**Table 2:** Factors of postpartum depression by using cox regression

Variables	Crude HR	95% CI	95% CI	*P*	Adjusted HR	95% CI	95% CI	*P*
**Sleep disorder**								
Without	Reference				Reference			
With	6.253	2.863	13.655	< 0.001	5.359	2.391	12.011	< 0.001
**Age group (years)**								
≦ 20	9.454	5.868	48.451	< 0.001	7.692	4.286	33.333	< 0.001
21–30	4.125	2.295	7.694	< 0.001	3.231	2.204	4.565	< 0.001
31–34	3.513	2.986	5.131	< 0.001	2.923	2.751	3.412	< 0.001
35–37	3.429	2.842	4.566	< 0.001	2.538	2.098	3.701	< 0.001
38–40	2.123	1.797	3.968	< 0.001	1.923	1.614	2.512	< 0.001
≧ 41	Referece				Reference			
**Number of births**								
1 (First-time birth)	4.712	2.895	6.502	< 0.001	3.345	2.121	4.995	< 0.001
≧ 2	Reference				Reference			
**Hypertension**								
Without	Reference				Reference			
With	0.000	-	-	0.302	0.000	-	-	0.600
**DM**								
Without	Reference				Reference			
With	0.485	0.066	3.575	0.478	0.852	0.112	6.479	0.877
**Hyperlipidemia**								
Without	Reference				Reference			
With	1.798	0.245	13.223	0.564	2.837	0.364	22.125	0.320
**COPD**								
Without	Reference				Reference			
With	0.000	-	-	0.581	0.000	-	-	0.726
**CKD**								
Without	Reference				Reference			
With	0.000	-	-	0.689	0.000	-	-	0.854
**IHD**								
Without	Reference				Reference			
With	0.000	-	-	0.533	0.000	-	-	0.755
**CHD**								
Without	Reference				Reference			
With	0.000	-	-	0.748	0.000	-	-	0.854
**Stroke**								
Without	Reference				Reference			
With	0.000	-	-	0.632	0.000	-	-	0.815
**Cancer**								
Without	Reference				Reference			
With	0.000	-	-	0.970	0.000	-	-	0.646
**Anxiety**								
Without	Reference				Reference			
With	4.696	1.118	19.724	0.035	2.423	1.273	7.424	0.043
**Depression**								
Without	Reference				Reference			
With	6.478	2.480	16.924	< 0.001	3.063	1.008	9.431	0.005
**Obesity**								
Without	Reference				Reference			
With	0.000	-	-	0.898	0.000	-	-	0.998
**Season**								
Spring (March-May)	Reference				Reference			
Summer (June-August)	0.580	0.206	1.629	0.301	0.513	0.181	1.459	0.211
Autumn (September-November)	1.058	0.446	2.513	0.898	0.867	0.361	2.087	0.751
Winter (December-Feburary)	0.324	0.088	1.195	0.091	0.306	0.082	1.134	0.076
**Urbanization level**								
1 (The highest)	0.860	0.312	2.365	0.769	0.957	0.345	2.654	0.932
2	0.682	0.252	1.845	0.451	0.781	0.286	2.132	0.630
3	0.846	0.211	3.381	0.813	0.861	0.214	3.461	0.833
4 (The lowest)	Reference				Reference			
**Insured premium (NT$)**								
< 18,000	Reference				Reference			
18,000–34,999	0.000	-	-	0.977	0.000	-	-	0.989
≧ 35,000	5.700	0.776	41.873	0.087	6.335	0.833	48.159	0.074

HR = hazard ratio, CI = confidence interval, Adjusted HR: Adjusted variables listed in the table.

The chances (risks) of developing PPD in pregnant women at the ages of ≤ 20 years, 21–30 years, 31–34 years, 35–37 years, and 38–40 years were 7.692-fold (*P* < 0.001), 3.231-fold (*P* < 0.001), 2.923-fold (*P* < 0.001), 2.538-fold (*P* < 0.001), and 1.923-fold (*P* < 0.001) increased compared with those ≥ 41 years. In addition, the chance (risk) of developing PPD in pregnant women with their first-time birth was 3.345-fold (*P* < 0.010) increased compared with pregnant women with their second birth. Moreover, the chance (risk) of developing PPD in pregnant women with anxiety was 2.423-fold (*P* = 0.043) increased compared with pregnant women without anxiety, and the chance (risk) of developing PPD in pregnant women with depression was 3.063-fold (*P* = 0.005) increased compared with women without depression.

Table 3 presents the stratified analyses of all variables to identify the fold increase in the risk of developing PPD in pregnant women with SD compared with pregnant women without SD. In the population of pregnant women aged 21–30 years after other factors were controlled, the chance (risk) of developing PPD in patients with SD was 8.593-fold (*P* = 0.001) increased compared with pregnant women without SD. In the population of pregnant women aged 31–34 years, the chance (risk) of developing PPD in patients with SD was 5.911-fold (*P* = 0.027) increased compared with pregnant women without SD. Among pregnant women with their first-time birth, the risk of developing PPD in patients with SD was 10.098-fold (*P* < 0.011) increased compared with pregnant women without SD.

**Table 3:** Factors of postpartum depression stratified by variables listed in the table by using cox regression

Sleep disorder	With			Without			Ratio	Adjusted HR	95% CI	95% CI	*P*
Variables	Event	PMs	Rate (per 10^5^ PMs)	Event	PMs	Rate (per 10^5^ PMs)					
**Total**	20	18,658.66	107.19	10	55,943.95	17.88	5.997	5.359	2.391	12.011	< 0.001
**Age group (years)**											
≦ 20	3	1,243.95	241.17	0	1,629.89	0.00	-	-	-	-	-
21–30	3	7,335.78	40.90	1	22,647.36	4.42	9.262	8.593	1.386	53.283	0.011
31–34	5	3,667.45	136.33	3	10,629.02	28.22	4.830	5.911	1.220	28.646	0.027
35–37	2	2,004.28	99.79	2	9,444.56	21.18	4.712	4.986	0.645	25.426	0.221
38–40	3	2,117.01	141.71	2	6,442.97	31.04	4.565	4.645	0.380	17.028	0.262
≧ 41	4	2,290.19	174.66	2	5,150.15	38.83	4.498	4.266	0.017	8.116	0.343
**Number of births**											
1 (First-time birth)	18	6,442.12	279.41	8	31,469.46	25.42	10.991	10.098	3.487	66.457	< 0.001
≧ 2	2	12,216.54	16.37	2	24,474.49	8.17	2.003	1.975	0.978	3.010	0.078
**Hypertension**											
Without	20	16,941.80	118.05	10	51,818.15	19.30	6.117	5.352	2.387	12.000	< 0.001
With	0	1,716.86	0.00	0	4,125.80	0.00	-	-	-	-	-
**DM**											
Without	19	17,107.84	111.06	10	52,477.96	19.06	5.828	5.169	2.282	11.710	< 0.001
With	1	1,550.82	64.48	0	3,465.99	0.00	-	-	-	-	-
**Hyperlipidemia**											
Without	19	18,189.00	104.46	10	54,980.47	18.19	5.743	5.189	2.282	11.710	< 0.001
With	1	469.66	212.92	0	963.48	0.00	-	-	-	-	-
**COPD**											
Without	20	17,921.33	111.60	10	55,001.65	18.18	6.138	5.352	2.387	12.000	< 0.001
With	0	737.33	0.00	0	942.30	0.00	-	-	-	-	-
**CKD**											
Without	20	18,464.45	108.32	10	55,259.15	18.10	5.985	5.352	2.387	12.000	< 0.001
With	0	194.21	0.00	0	684.80	0.00	-	-	-	-	-
**IHD**											
Without	20	18,014.99	111.02	10	54,429.10	18.37	6.043	5.352	2.387	12.000	< 0.001
With	0	643.67	0.00	0	1,514.85	0.00	-	-	-	-	-
**CHD**											
Without	20	18,498.69	108.12	10	55,523.08	18.01	6.003	5.352	2.387	12.000	< 0.001
With	0	159.97	0.00	0	420.87	0.00	-	-	-	-	-
**Stroke**											
Without	20	18,232.62	109.69	10	55,090.86	18.15	6.043	5.352	2.387	12.000	< 0.001
With	0	426.04	0.00	0	853.09	0.00	-	-	-	-	-
**Cancer**											
Without	20	18,563.13	107.74	10	55,753.07	17.94	6.007	5.258	2.277	12.135	< 0.001
With	0	95.53	0.00	0	190.88	0.00	-	-	-	-	-
**Anxiety**											
Without	19	17,998.73	105.56	9	50,515.04	17.82	5.925	4.447	0.918	10.879	0.621
With	1	659.93	151.53	1	4,428.92	22.58	6.711	6.032	2.592	14.039	< 0.001
**Depression**											
Without	16	17,293.65	92.52	9	53,880.66	16.70	5.539	4.829	0.114	6.039	0.853
With	4	1,365.01	293.04	1	2,063.29	48.47	6.046	6.652	2.750	16.088	< 0.001
**Obesity**											
Without	20	18,613.67	107.45	10	55,890.63	17.89	6.005	5.352	2.387	12.000	< 0.001
With	0	44.99	0.00	0	53.32	0.00	-	-	-	-	-
**Season**											
Spring (March-May)	7	3,707.07	188.83	2	13,228.77	15.12	12.490	6.301	1.214	32.704	0.028
Summer (June-August)	4	4,650.99	86.00	2	14,620.77	13.68	6.287	9.060	1.422	57.171	0.020
Autumn (September-November)	8	6,137.54	130.35	4	15,093.64	26.50	4.918	4.085	1.128	14.798	0.032
Winter (December-Feburary)	1	4,163.06	24.02	2	13,001.27	15.38	1.562	1.803	0.038	17.081	0.890
**Urbanization level**											
1 (The highest)	6	4,965.34	120.84	4	18,074.03	22.13	5.460	4.352	1.055	17.950	0.042
2	6	7,927.42	75.69	5	24,438.70	20.46	3.699	2.468	0.676	9.020	0.171
3	2	2,087.67	95.80	1	5,090.88	19.64	4.877	4.341	0.390	48.277	0.232
4 (The lowest)	6	3,678.23	163.12	0	8,340.34	0.00	-	-	-	-	-
**Insured premium (NT$)**											
< 18,000	20	18,189.49	109.95	9	54,323.25	16.57	6.637	5.851	2.532	13.519	< 0.001
18,000–34,999	0	400.19	0.00	0	1,261.30	0.00	-	-	-	-	-
≧ 35,000	0	68.98	0.00	1	359.40	278.24	0.000	0.000	-	-	0.990

PMs = Person-months; Adjusted HR = Adjusted Hazard ratio: Adjusted for the variables listed in Table 3.; CI = confidence interval.

Table 4 presents the stratified analyses on postpartum time (≤ 6 weeks, 6–12 weeks, and > 12 weeks). Regardless of postpartum time (≤ 6 weeks, 6–12 weeks, and > 12 weeks), the risks of developing PPD in pregnant women with SD were increased, by 5.461-fold (*P* < 0.001), 3.490-fold (*P* = 0.010), and 3.416-fold (*P* = 0.015), respectively, compared with pregnant women without SD.

**Table 4:** Factors of postpartum depression stratified by differ times of postpartum in the table by using cox regression

Sleep disorder	With			Without			Ratio	Adjusted HR	95% CI	95% CI	*P*
Variables	Event	PMs	Rate (per 10^5^ PMs)	Event	PMs	Rate (per 10^5^ PMs)					
**Total**	20	18,658.66	107.19	10	55,943.95	17.88	5.997	5.359	2.391	12.011	< 0.001
**Times of postpartum**											
≦ 1.5 months (6 weeks)	12	1,034.79	1,159.66	3	1,241.92	241.56	4.801	5.461	1.495	19.952	< 0.001
> 1.5 months (6 weeks), ≦ 3 months (12 weeks)	4	2,431.35	164.52	2	4,111.42	48.64	3.382	3.490	1.282	7.197	0.010
> 3 months (12 weeks)	4	15,192.52	26.33	5	50,590.61	9.88	2.664	3.416	1.072	5.110	0.015

PMs = Person-months; Adjusted HR = Adjusted Hazard ratio: Adjusted for the variables listed in Table 3.; CI = confidence interval.

**Figure 3:**
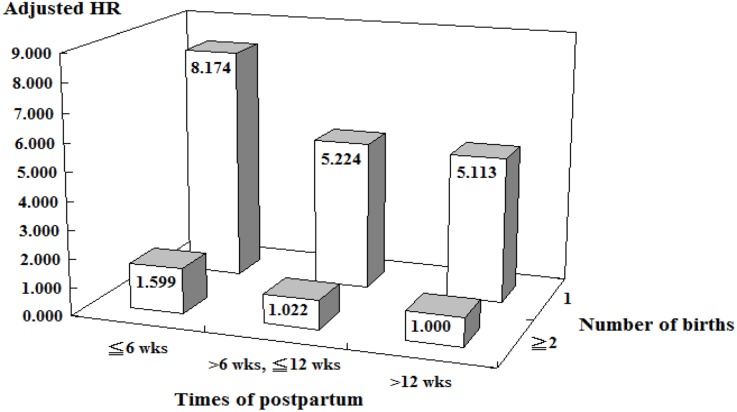
Association between sleep disorder and postpartum depression stratified by times of postpartum and number of births by using Cox regression wks = weeks, Adjusted HR = Adjusted Hazard ration: Adjusted for the variables listed in Table 3 Interaction (Times of postpartum × Number of births) *P* < 0.001.

Figure 3 presents the cross analyses of pregnancy parity and postpartum time. The risk of developing PPD in pregnant women with their first-time pregnancy within postpartum 6 weeks was the highest and was 8.174-fold increased compared with pregnant women with their second (or greater) pregnancy at postpartum 12 weeks (or longer).

## DISCUSSION

SD are a risk factor for developing PPD in women in Taiwan. SD increased the risk of developing PPD in women (the risk in cases with SD was 5.359-fold increased compared with cases without SD). In addition, regardless of postpartum time (≤ 6 weeks, 6–12 weeks, and > 12 weeks), the chance (risk) of developing PPD was greater than 3.416-fold increased in pregnant women with SD compared with pregnant women without SD. Previous studies revealed that patients with SD are likely aware of bad health conditions or negative emotions, such as working under great stress [15, 21]. Moreover, the majority of studies also indicate that pregnant women are under great stress with their first birth due to physical and mental changes, expectations of newborns, and uncertainty of family and social welfare support [22]. As a result, this study demonstrates that pregnant women experiencing their first birth exhibit increased risks of developing PPD. Reduced sleep quality during pregnancy might be an important risk factor for developing PPD. Therefore, persistent relief of SD is very important.

SD are a defined as difficulty falling asleep, light sleep, and frequent periods of being awake at night with symptoms persisting for at least one month. SD are a common disease and can affect the quality of life of a normal individual [23]. Most current articles focus on the association between SD and pressure, shift work, and reproductive dysfunction as study topics [24–27] but did not perform a similar analysis on the risk of developing PPD caused by SD. This study was the first study in Taiwan relevant to this topic.

Previous studies noted that poor sleep quality was highly associated with depression [28, 29]. In addition, some studies investigated the development of mood disorders in pregnant women during the postpartum period using the Pittsburgh Sleep Quality Index. Poor sleep quality in late pregnancy increased the risk of depression recurrence. In particular, poor subjective sleep quality was highly associated with PPD symptoms [14, 30]. Furthermore, some scholars utilized wrist actigraphy and self-report surveys to study the association between sleep quality and PPD symptoms using longitudinal study surveys. The results revealed that poor sleep quality could be a risk factor for developing PPD and demonstrated that poor sleep quality in women in the early postpartum period was associated with symptoms of depression [31]. The above study results were similar to those in this study. Data in this study even indicated that SD increased the risk of developing PPD (regardless of postpartum time) and could be further used as an assessment item in the prenatal examinations of pregnant women.

This study demonstrated that among pregnant women with SD, the risk of developing PPD was the highest in pregnant women experiencing their first birth or at a young pregnant age, which is similar to findings in other cross-sectional studies. Studies noted that women experiencing their first birth and less than 25 years of age exhibited an increased risk of developing PPD if they had poor sleep quality or insomnia complaints during pregnancy [28, 32]. It is recommended that SD be considered an important predictive factor affecting PPD. During prenatal examinations of pregnant women, the sleep conditions of women experiencing their first birth and less than 25 years of age should be actively assessed to reduce the risk of developing PPD.

In addition, this study demonstrated that the risk of developing PPD in pregnant women with a history of depression was 3.063-fold increased compared with women without a history of this disease. These results were similar to those in studies in other countries demonstrating that SD in late pregnancy increased the recurrence of postpartum major depression (PPMD), especially in cases with a history of major depression [14]. The possible reason for this finding was that pregnant women in early pregnancy already began to experience different challenges of pregnancy and were persistently subjected to various pressures. If depression developed before pregnancy, the possibility of developing PPD greatly increased. In addition, poor sleep quality in late pregnancy was associated with recurrence. Furthermore, this study revealed another phenomenon: the risk of developing PPD in pregnant women with a history of anxiety was 2.423-fold increased compared with pregnant women without a history of anxiety. However, no relevant study is available for comparison in Taiwan or other countries; thus, further studies in this field should be performed.

This study has the following limitations. First, the health insurance data do not include the variable of the severity of SD. Therefore, relevant data are not available to discuss the severity of SD. Second, the associations between social demographic factors, such as age, number of births, and disease history, and PPD were analyzed. However, other psychological and social variables, such as potential influencing factors including the family support level and the education level of the pregnant women, might also affect PPD; however, these variables could not be further investigated in this study.

## MATERIALS AND METHODS

### Data sources

This study performed analyses using data from the NHIRD of Taiwan from between January 1, 2000 and December 31, 2010. National health insurance was implemented in Taiwan in 1995. The NHIRD records the medical information of all insured, with a coverage rate of greater than 99% of the Taiwanese population. Therefore, we obtained data from the NHIRD to investigate the association between SD and PPD during this 10-year period. The diagnosis used in the NHIRD is the International Classification of Diseases, Ninth Revision, Clinical Modification (ICD-9-CM).

### Study design and participant sampling

This study adopted the follow-up study design. New cases of pregnant women who were diagnosed with SD during pregnancy in the database were screened and selected as the study subjects in the study subject group. The study period was between January 1, 2000 and December 31, 2010. For comparison with the subject group, a control group with twice the number of subjects as the subject group with matched age, income, and number of births was selected (pregnant women without a diagnosis of SD).

### Occurrence

All study participants were observed from the beginning of pregnancy until the development of PPD (ICD-9-CM 648.4) or until the end of this study on December 31, 2010.

### Age and comorbidities

Study subjects were divided into 6 age groups (≤ 20 years, 21–30 years, 31–34 years, 35–37 years, 38–40 years, and ≥ 41 years). Comorbidities included hypertension (ICD-9-CM: 401–405), diabetes mellitus (DM; ICD-9-CM: 250), hyperlipidemia (ICD-9-CM: 272), chronic obstructive pulmonary disease (COPD; ICD-9-CM: 490–496), chronic kidney disease (CKD; ICD-9-CM: 585), ischemic heart disease (IHD; ICD-9-CM:410–414), congestive heart disease (CHD; ICD-9-CM:428–429), stroke (ICD-9-CM: 430–438), cancer (ICD-9-CM: 140–208), anxiety (ICD-9-CM: 300.00), and depression (ICD-9-CM 296.2–296.3, 300.4).

### Statistical analyses

All analyses were performed using SPSS 22 software (SPSS, Inc., Chicago, IL, USA). Basic descriptive statistics included percentage, mean value, and standard deviation. Relevant study variables included age, urbanization, income, season, comorbidity, and number of births. In addition, comparisons of categorical variables (with and without SD) were performed using the Χ^2^ test. The differences between two groups were expressed using the hazard ratio (HR) and 95% confidence interval (CI). *P* < 0.05 was used as the standard for determining significance.
